# L‐Carnitine Prevents Lipopolysaccharide‐Induced Inflammatory Response, Microglia Activation and Neuronal Damage in Cerebral Cortex, as Well as Neuromotor Development Delay in Neonatal Glutaryl‐CoA Dehydrogenase Deficient (*Gcdh^−/−^*) Mice

**DOI:** 10.1002/jimd.70253

**Published:** 2026-09-20

**Authors:** Ediandra Tissot Castro, Ângela Zanatta, Rafael Teixeira Ribeiro, Diorlon Nunes Machado, Andrey Vinicios Soares Carvalho, Ângela Beatris Zemniaçak, Sâmela de Azevedo Cunha, Tailine Quevedo Tavares, Manuela Bianchin Marcuzzo, Larissa Daniele Bobermin, Carlos Alexandre Netto, Alexandre Umpierrez Amaral, Carmen Regla Vargas, Guilhian Leipnitz, Moacir Wajner

**Affiliations:** ^1^ PPG Ciências Biológicas: Bioquímica, Departamento de Bioquímica, ICBS, UFRGS Porto Alegre Brazil; ^2^ PPG Atenção Integral à Saúde (UNICRUZ/URI‐Erechim/UNIJUÍ), URI Erechim Brazil; ^3^ Serviço de Genética Médica, Hospital de Clínicas de Porto Alegre Porto Alegre Brazil; ^4^ PPG Ciências Farmacêuticas, UFRGS Porto Alegre Brazil

**Keywords:** *Gcdh* ^*−/−*^ mice, glutaric acid, Glutaric aciduria type I, histopathology, L‐carnitine, neurodevelopment, neuroinflammation

## Abstract

Patients with glutaric acidemia type I (GA I) frequently manifest with acute encephalopathy usually triggered by infections and by progressive neurological deterioration. Since the pathogenesis of the brain damage in GA I during inflammatory processes is poorly established, we investigated biomarkers of inflammatory response and neural damage in the cerebral cortex of wild type (WT) and glutaryl‐CoA dehydrogenase (GCDH) deficient mice (*Gcdh* ^*−/−*^) receiving acute lysine and lipopolysaccharide (LPS) administration to induce inflammation. Neuromotor development reflexes and the neuroprotective effects of L‐carnitine (Carn), whose potent anti‐inflammatory properties have been recently described, were also evaluated. LPS increased the gene expression of most parameters of the inflammatory response, which were normalized or attenuated by Carn both in WT and *Gcdh* ^*−/−*^ mice. Importantly, gene expression of TNF‐α, IL‐6, NFkB, IκBα, COX‐2 and VEGF, and heme oxygenase‐1 content were significantly increased in LPS‐treated *Gcdh* ^*−/−*^ relatively to the WT mice. Furthermore, the neuronal biomarker protein NeuN was reduced, whereas the number of vacuoles and neurodegenerative cells were increased in the LPS‐treated *Gcdh* ^*−/−*^ mice, indicating neuronal damage and loss in these animals. Furthermore, the righting and the gait reflexes were altered in the *Gcdh* ^*−/−*^ mice, reflecting impairment of neuromotor development. Finally, Carn prevented LPS‐induced inflammation, neuronal damage, vacuolation, increased neurodegenerative cells and the righting reflex. Our findings suggest that inflammation plays an important role in the pathogenesis of the neurological alterations following infectious/inflammatory processes in GA I patients and that higher doses of Carn should be considered during these episodes.

## Graphical abstract

L‐Carnitine abrogates LPS‐induced brain inflammatory response leading to neurodegeneration and neurodevelopment delay in a genetic murine model of GAI.

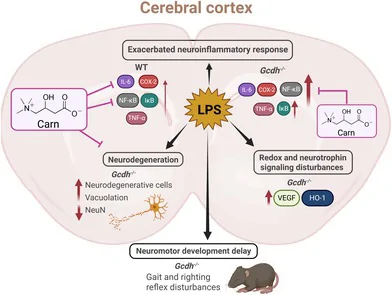

## 1 Introduction

Glutaric acidemia type I (GA I OMIM #231670) is caused by deficient activity of glutaryl‐CoA dehydrogenase (GCDH) [1] with a global incidence ranging from 1/30 000 to 1/100 000 newborns [2, 3, 4]. Reduced GCDH activity causes accumulation of glutaric acid (GA) and 3‐hydroxyglutarate (3OHGA) [5, 6, 7], which have been shown to provoke brain damage [8, 9, 10, 11].

Neurological symptoms including dystonia, hypotonia, spasticity, seizures, cognitive impairment, and encephalopathic crises occurring in the first years of life are common, as well as cerebral abnormalities mainly in the basal ganglia and cerebral cortex [12, 13, 14, 15, 16]. Early diagnosis by neonatal screening and rapid initiation of treatment significantly reduce morbidity and mortality [17].

Two groups of GA I patients were described. The high excretors present high levels of GA in the urine (≥ 100 mmol/mol creatinine), and the low excretors low levels of this organic acid (< 100 mmol/mol creatinine, LE) [9, 18, 19, 20].

GA I therapy is based on a low‐lysine (Lys) diet and L‐carnitine supplementation [17]. However, patients diagnosed by newborn screening and submitted to early treatment may manifest encephalopathic crises but to a lesser degree, fine motor deficits [18, 21], and renal complications [22]. Thus, considering that treatment remains insufficient for many patients, it is crucial to unravel the underlying disease mechanisms of brain damage to develop novel and effective therapies for GA I.

A genetic knockout (KO) murine model of GA I (*Gcdh* ^*−/−*^) was developed to facilitate the investigation of the pathogenesis of this disorder, and later improved by feeding the animals with high protein or Lys overload [23, 24]. In this particular, in vitro and in vivo studies using *Gcdh* ^*−/−*^ mice revealed disturbances of bioenergetics, oxidative stress, and excitotoxicity in the cerebral cortex and striatum [25, 26, 27, 28, 29, 30, 31, 32, 33, 34]. However, the role of systemic inflammation and increased neuroinflammatory response in the neonatal brain and their damaging consequences to the brain are still poorly known in this GA I genetic model and also in GA I patients.

A previous study testing the influence of intraperitoneal administration of lipopolysaccharide (LPS) in the genetic knockout (KO) murine model of GA I (*Gcdh* ^*−/−*^) showed alterations of redox homeostasis in the brain of 30‐day‐old animals [35]. Thus, considering that systemic infections are accompanied by an increased inflammatory response and usually precipitate episodes of acute encephalopathy in GA I patients, it is hypothesized that systemic inflammation associated with neuroinflammation may trigger still unknown deleterious mechanisms leading to cortical and striatal injuries in patients. The present work investigated the mRNA expression of pro‐inflammatory cytokines and other inflammatory biomarkers, as well as the signaling pathways underlying the inflammatory response in the cerebral cortex of WT and *Gcdh* ^*−/−*^ mice subjected to acute Lys overload to increase the toxic organic acid derivatives GA and 3OHGA and intraperitoneal LPS to induce systemic inflammation. Microglial activation, vacuolation, neuronal death and degenerative neural cells were also examined under these conditions, as well as neuromotor development. Finally, the neuroprotective role of L‐carnitine (Carn) treatment, particularly its anti‐inflammatory properties [36, 37, 38, 39, 40], was also evaluated.

## 2 Material and Methods

### 2.1 Chemicals, Animals, and Ethical Statement

All chemicals were obtained from Sigma–Aldrich (St. Louis, MO, USA), unless otherwise specified. Lys (Sigma–Aldrich #L5501), LPS (Sigma–Aldrich #L4005) and Carn (Sigma–Aldrich #C0283) were diluted in phosphate‐buffered saline (PBS), and the pH was adjusted to 7.4.

Male and female wild‐type (WT) and glutaryl‐CoA dehydrogenase–deficient (*Gcdh* ^*−/−*^) mice (C129SvEv background) were housed at the Experimental Animal Facility of the Hospital de Clínicas de Porto Alegre (Brazil), with ad libitum access to water and standard chow (20% protein).

All procedures complied with Brazilian Federal Law No. 11.794/2008 and CONCEA guidelines and were approved by the Animal Experimentation Ethics Committee of the Hospital de Clínicas de Porto Alegre (protocol 20230483).

### 2.2 Lysine (Lys), Lipopolysaccharide (LPS) and L‐Carnitine (Carn) Administration

**FIGURE 1:**
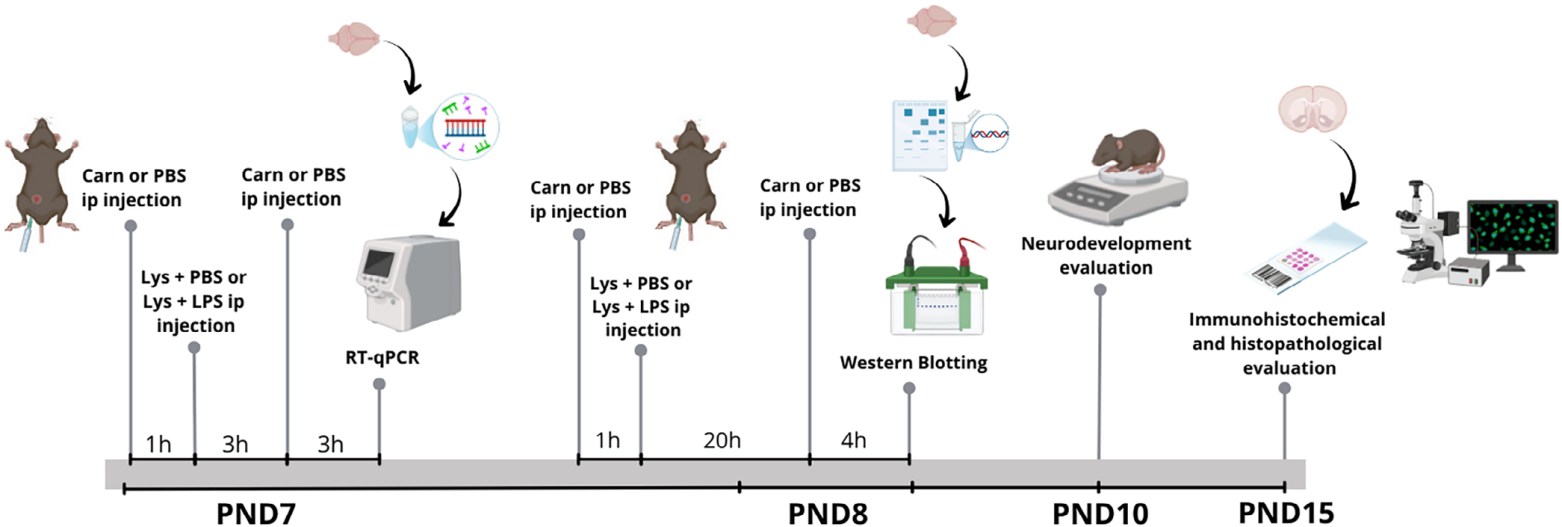
Timeline showing the experimental design and the biochemical, immunohistochemical and neurodevelopmental tests carried out from postnatal day (PND) 7 to 15. Male and female wild type (WT) and *Gcdh* ^*−/−*^ mice were used in the experiments. Phosphate buffered saline (PBS), Lysine (Lys), lipopolysaccharide (LPS), and L‐carnitine (Carn) were injected intraperitoneally (ip).

A total of 127 neonatal WT and *Gcdh* ^*−/−*^ were used in the experiments. They were randomly separated, weighed, marked, and divided into six groups: WT mice: (1) PBS + Lys + PBS; (2) PBS + Lys + LPS; (3) Carn + Lys + LPS; *Gcdh* ^−/−^ mice: (4) PBS + Lys + PBS; (5) PBS + Lys + LPS; (6) Carn + Lys + LPS. The experimental timeline from postnatal day (PND) 7 to PND15 is shown in Figure 1.

On PND7, all animals received an acute intraperitoneal (i.p.) injection of Lys (4 μmol/g), which leads to increased production of GA and 3OHGA in the brain of *Gcdh* ^*−/−*^ animals [24, 41, 42]. To induce systemic and CNS inflammatory responses, groups 2, 3, 5, and 6 additionally received LPS (5 mg/kg, i.p.) [35, 43], while groups 1 and 4 received PBS (vehicle). Groups 3 and 6 were pretreated with two injections of Carn (100 mg/kg, i.p.) [37, 44] the first injection 1 h before Lys injection and the second 1 h before LPS administration; the remaining groups received PBS (vehicle). For RT‐qPCR analyses, a second Carn injection was administered 3 h after Lys/LPS treatment, whereas for western blotting and immunofluorescence experiments, the second Carn dose was given 20 h after Lys/LPS administration.

### 2.3 Tissue Preparation

The animals were decapitated after being deeply anesthetized with isoflurane, and the cerebral cortex was separated, properly identified, and prepared according to each technique.

#### 2.3.1 Quantitative RT‐PCR

To investigate gene expression via quantitative PCR, we utilized mice at PND7 that had been euthanized 6 h after LPS and 3 h after Carn administration. Total RNA was isolated from the cerebral cortex with TRI Reagent (Sigma–Aldrich, #T9424, USA). The integrity and concentration of the isolated RNA were determined spectrophotometrically by the 260/280 nm absorbance ratio. Following this, cDNA was synthesized from 2 μg of total RNA using the High‐Capacity cDNA Reverse Transcription Kit (Thermo Fisher Scientific, #4368814). The relative mRNA quantification of IL‐6 (Thermo Fisher Scientific, #Mm00446190_m1), pro‐inflammatory cytokine TNF‐α (Thermo Fisher Scientific, #Mm00443258_m1), inflammatory enzyme COX‐2 (Thermo Fisher Scientific, #Mm00478374_m1), transcription factor NFκB (Thermo Fisher Scientific, #Mm00476361_m1), IκBα, the NFκB regulatory protein (Thermo Fisher Scientific, #Mm00477798_m1), and vascular endothelial growth factor VEGF (Thermo Fisher Scientific, #Mm00437306_m1) was measured by real‐time PCR with specific TaqMan gene expression assays (Thermo Fisher Scientific). Normalization was performed against the housekeeping gene β‐actin (Thermo Fisher Scientific, #Mm02619580_g1), and the data were analyzed according to the 2^−ΔΔCt^ method [45].

#### 2.3.2 Western Blot Analysis

For immunoblotting, mice were euthanized 20 h after LPS administration (PND8), and the cerebral cortex was homogenized in RIPA buffer supplemented with protease and phosphatase inhibitors. Homogenates were centrifuged at 10 000 × g for 10 min, and protein concentrations in the supernatants were determined. Samples were denatured in 2× Laemmli buffer (Bio‐Rad, #1610737) containing 10% β‐mercaptoethanol and heated at 70°C for 10 min.

Cerebral cortex samples, each containing 30 μg of protein per well, were applied to commercial polyacrylamide gels (Thermo Fisher Scientific, #NP0323BOX) and then transferred to nitrocellulose membranes using a Power Blotter System (Thermo Fisher Scientific) as previously described [46]. Protein loading and transfer efficiency were verified by Ponceau S staining. Membranes were blocked with 5% bovine serum albumin in TTBS and incubated overnight at 4°C with anti‐HO‐1 antibody (mouse, 1:1000; Thermo Fisher Scientific, #MA1‐112). After washing, membranes were incubated for 2 h with HRP‐conjugated secondary antibody (anti‐mouse, 1:5000; Santa Cruz, #sc‐2031). Immunoreactive bands were visualized using enhanced chemiluminescence (Pierce ECL Western Kit; Thermo Fisher Scientific, #32106) on a ChemiDoc MP Imaging System (Bio‐Rad). Densitometric analysis was performed using Fiji software. β‐Actin (mouse, 1:1000; Cell Signaling, #4967) was used as a loading control, and results were expressed as the ratio of target protein to β‐actin (*n* = 4 animals per group).

#### 2.3.3 Immunohistochemical Analysis

For immunohistochemistry, mice were anesthetized on PND15 and transcardiacly perfused with 0.9% saline containing 0.16% sodium citrate, followed by 4% paraformaldehyde (PFA). Brains were removed, post‐fixed in 4% PFA for 24 h, and sectioned coronally (35 μm) using a vibratome (VT1000S; Leica). Three independent sessions containing cerebral cortex sections from three animals per group were analyzed. Immunolabeling was performed on free‐floating sections as previously described [34, 47]. Sections were permeabilized with PBS–Triton, blocked with 5% albumin in PBS–Triton, and incubated overnight at 4°C with primary antibodies against NeuN (mouse, 1:500; Millipore) and Iba1 (rabbit, 1:500; Wako). After washing, sections were incubated for 2 h with the appropriate secondary antibodies (Alexa Fluor 488 anti‐mouse and Alexa Fluor 546 anti‐rabbit; Invitrogen). Sections were mounted with Fluoroshield (Sigma–Aldrich), and images were acquired using an Olympus FV300 confocal microscope. Quantification of NeuN‐positive cells and Iba1 fluorescence intensity was performed using Fiji software.

#### 2.3.4 Histopathological Studies

Histopathological analyses were performed in the brain of WT and *Gcdh* ^*−/−*^ mice on PND15, utilizing hematoxylin and eosin (HE) staining. Following deep anesthesia with isoflurane, animals were euthanized, and brains were removed and fixed in 4% paraformaldehyde (PFA) for 24 h. Tissues were then dehydrated through graded ethanol and xylene, embedded in paraffin, and sectioned as previously described [34, 48]. Coronal sections (7 μm thick) at Bregma 1.20 mm [49] were obtained using a rotary microtome (Leica RM‐2125) and mounted on glass slides, followed by HE staining (Sigma–Aldrich).

Histopathological evaluation was performed in the primary motor cortex (M1) using light microscopy (Nikon Eclipse E‐600) at 400× magnification. The number of degenerating neurons and vacuoles was quantified using ImageJ software. Five sections per animal (one field per section) were analyzed, and mean values were calculated. Degenerating neurons were identified by cell body deformation or the presence of karyolytic, pyknotic, or karyorrhexic nuclei [48, 50]. Data were expressed as the percentage of degenerating cells, calculated as (mean degenerating cells/mean total cells) × 100.

### 2.4 Neurodevelopmental Assessment

Neurodevelopmental reflexes were assessed on PND10 by a blinded evaluator between 13:00 and 16:00, and body weight gain was also recorded.

#### 2.4.1 Righting Reflex

This test evaluates motor function and coordination by placing the animal in a supine position and measuring the latency to return to the prone position; three trials are performed per animal, with a maximum cumulative latency of 15 s [51].

#### 2.4.2 Gait

To assess gait, animals were placed at the center of a 15‐cm‐diameter circle, and the latency to exit the circle with both forepaws was recorded; three trials were performed, with a maximum latency of 30 s [52].

#### 2.4.3 Negative Geotaxis

This test assesses sensorimotor and vestibular function by placing pups head‐down on a 45° inclined surface and recording the latency to orient upward and complete a 180° turn; three trials were performed per animal, with a maximum latency of 30 s per trial [53].

### 2.5 Statistical Analysis

Results are expressed as mean ± standard deviation (SD). The sample size was determined using the Minitab software (version 16). We assumed a target power of 0.9, a standard deviation of 10% to 20% and a difference of 30%. The number of animals (*n*) used in each experiment is indicated in the figure legends. Data normality were evaluated using the Shapiro–Wilk test, and homogeneity of variances was assessed by Bartlett's test. Statistical analyses were performed using two‐way ANOVA followed by Tukey's post hoc test, considering genotype (WT or *Gcdh* ^*−/−*^), treatment, and their interaction as factors. *F* values are reported when significant. A value of *p* < 0.05 was considered statistically significant. All analyses were conducted using GraphPad Prism software (version 10.1.2).

## 3 Results

### 3.1 Carn Prevents LPS‐Induced Alterations of mRNA Expression of TNF‐α and IL‐6 Pro‐Inflammatory Cytokines in Cerebral Cortex of 7‐Day‐Old WT and Gcdh^−/−^ Mice

**FIGURE 2:**
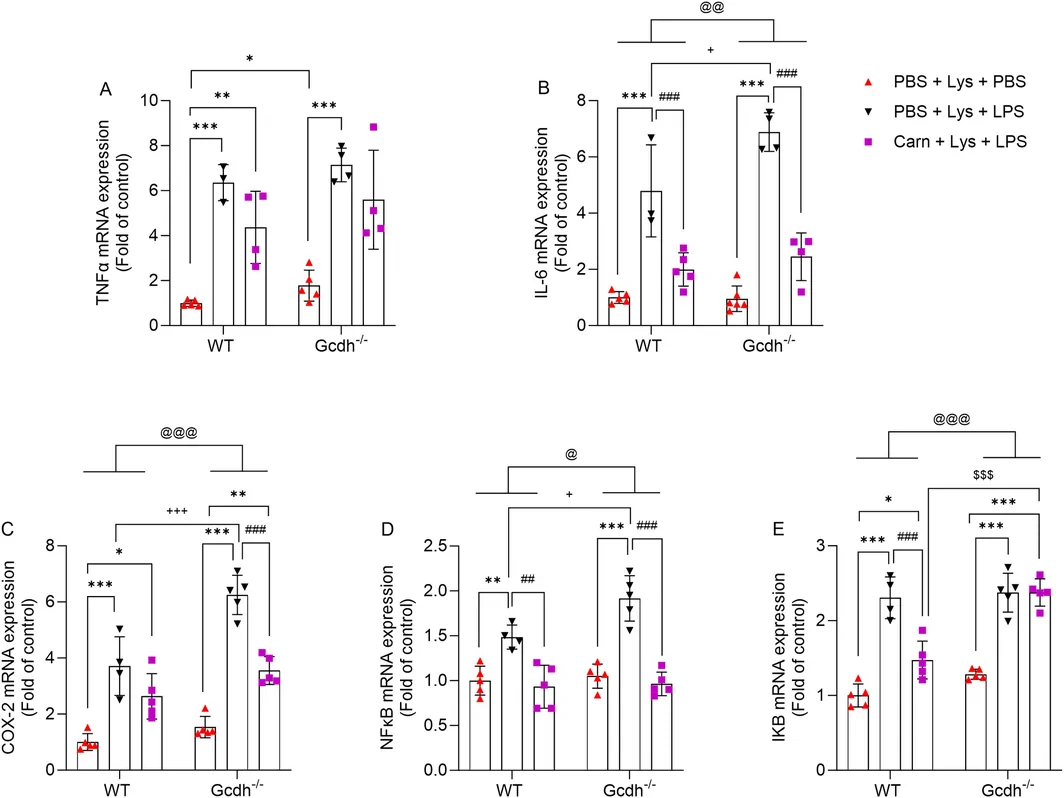
Alterations of the mRNA expression of TNF‐α (A), IL‐6 (B), COX‐2 (C), NFκB (D), and IκB (E) in the cerebral cortex of 7‐day‐old WT and *Gcdh* ^*−/−*^ mice. All animals were administered ip with Lys (4 μmol/g), whereas some with LPS (5 mg/kg), and others with LPS plus Carn (100 mg/kg). Data are presented as mean ± standard deviation (SD), with *n* = 3–6 animals per group. Statistical analysis was performed using two‐way ANOVA followed by the Tukey's post hoc test (*p < 0.05, **p < 0.01, ***p < 0.001, different from PBS + Lys + PBS; ^##^ *p* < 0.01, ^###^ *p* < 0.001, difference between PBS + Lys + LPS and Carn + Lys + LPS; ^$$$^ *p* < 0.001 difference between WT and *Gcdh* ^*−/−*^ mice within the Carn + Lys + LPS treatment group; ^+^ *p* < 0.05, ^+++^ *p* < 0.001 difference between WT and *Gcdh* ^*−/−*^ in LPS treated animals; ^@^ *p* < 0.05, ^@@^ *p* < 0.01, ^@@@^ *p* < 0.001difference between WT and *Gcdh* ^*−/−*^).

First, we determined the mRNA levels of pro‐inflammatory markers in the brain of WT and *Gcdh* ^*−/−*^ mice. We observed that TNF‐α expression was higher in *Gcdh* ^*−/−*^ relative to WT mice (Figure 2A). It can also be seen that LPS treatment highly increased the gene expression of TNF‐α (Figure 2A) and IL‐6 (Figure 2B) and that Carn markedly reduced the LPS‐induced increase of mRNA levels of these pro‐inflammatory cytokines in the *Gcdh* ^*−/−*^ and attenuated in the WT mice (TNF‐α: Treatment *F*_(2,19)_ = 44.78, *p* < 0.0001; Interaction *F*_(2,19)_ = 0.095, *p* = 0.9101; Genotype *F*_(2,19)_ = 3.68, *p* = 0.0702; IL‐6: Treatment *F*_(2,21)_ = 92.41, *p* < 0.0001; Interaction *F*_(2,21)_ = 4.49, *p* = 0.0238; Genotype *F*_(1,21)_ = 8.10, *p* = 0.0097).

### 3.2 Carn Modulates LPS‐Induced Inflammatory Signaling in Cerebral Cortex of 7‐Day‐Old Mice

We next investigated some inflammatory pathways as potential targets associated with LPS‐induced neuroinflammation, as well as Carn anti‐inflammatory effects. We measured the mRNA expression of COX‐2, NFkB and IκB in the cerebral cortex of WT and *Gcdh* ^*−/−*^ mice submitted to LPS acute administration and observed a significant increase of the biomarkers COX‐2 (Figure 2C) (Treatment *F*_(2,23)_ = 76.21, *p* < 0.0001; Interaction *F*_(2,23)_ = 6.15, *p* = 0.0073; Genotype *F*_(1,23)_ = 30.03, *p* < 0.0001), NFkB (Figure 2D) (Treatment *F*_(2,23)_ = 46.30, *p* < 0.0001; Interaction *F*_(2,23)_ = 3.427, *p* = 0.0498; Genotype *F*_(1,23)_ = 6.16, *p* = 0.0208) and IκBα (Figure 2E) (Treatment *F*_(2,23)_ = 81.08, *p* < 0.0001; Interaction *F*_(2,23)_ = 10.33, *p* = 0.0006; Genotype *F*_(1,23)_ = 28.93, *p* < 0.0001) in the brain of the *Gcdh* ^*−/−*^ mice compared to the WT animals, particularly in those injected with LPS. Furthermore, Carn attenuated the altered mRNA expression of COX‐2 (Figure 2C) and normalized NFkB gene expression (Figure 2D) but had no effect on IκB mRNA expression (Figure 2E).

### 3.3 Carn Attenuates LPS‐Induced Increase of HO‐1 Protein Content in Cerebral Cortex of 7‐Day‐Old Gcdh^−/−^ Mice

**FIGURE 3:**
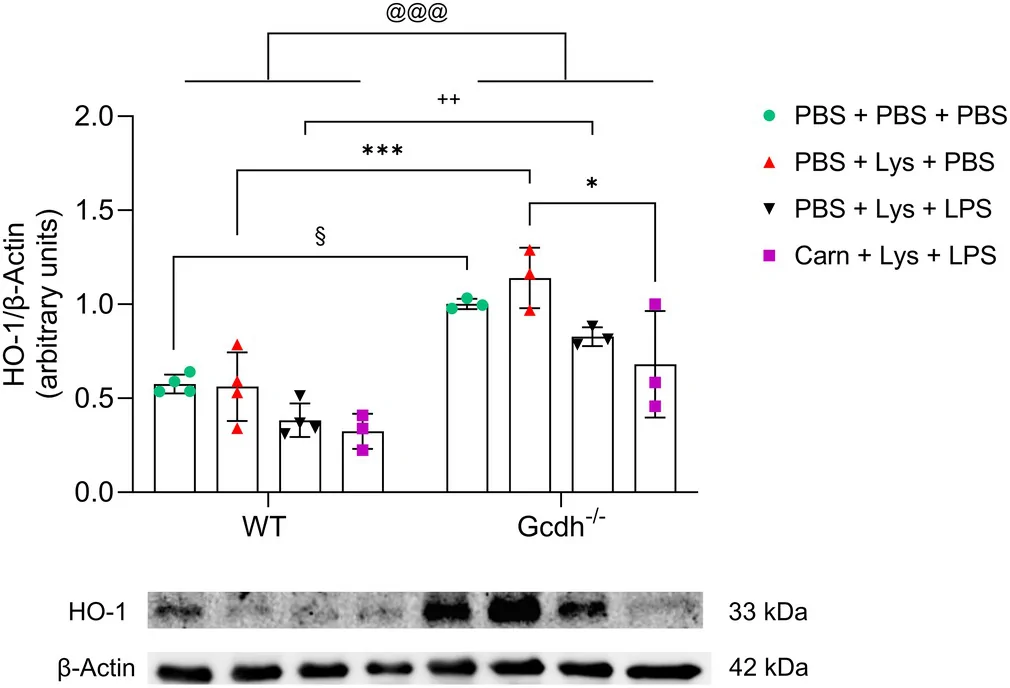
Increased heme oxygenase‐1 (HO‐1) protein levels in the cerebral cortex of 7‐day‐old *Gcdh* ^*−/−*^ mice. All animals were administered ip with Lys, whereas some with LPS and others with LPS plus Carn. Statistical analysis was conducted using two‐way analysis of variance (ANOVA), followed by Tukey's post hoc test. Results are presented as mean ± SD, with *n* = 3–5 animals per group. (*p < 0.05, ***p < 0.001 different from PBS + Lys + PBS; ^§^ *p* < 0.05 difference between WT and *Gcdh* ^*−/−*^ mice within the PBS + PBS + PBS treatment group; ^++^ *p* < 0.01 difference between WT and *Gcdh* ^*−/−*^ in LPS treated animals, ^@@@^ *p* < 0.001 difference between WT and *Gcdh* ^*−/−*^).

Since inflammatory responses are usually associated with a modulation of antioxidant defenses [54], we measured the levels of HO‐1 in the cerebral cortex. It can be seen in Figure 3 that HO‐1 protein level was markedly increased in the cerebral cortex of *Gcdh* ^*−/−*^ relative to WT mice treated or nontreated with LPS (Treatment *F*_(3,19)_ = 8.65, *p* = 0.0008; Interaction *F*_(3,19)_ = 0.73, *p* = 0.5493; Genotype *F*_(1,19)_ = 69.32, *p* < 0.0001). Furthermore, LPS did not further increase HO‐1 expression in both variants, whereas Carn partly prevented HO‐1 protein content in LPS‐injected *Gcdh* ^*−/−*^ mice.

### 3.4 Increased Vascular Endothelial Growth Factor (VEGF) Gene Expression in Cerebral Cortex of 7‐Day‐Old LPS‐Injected Gcdh^−/−^ Mice

**FIGURE 4:**
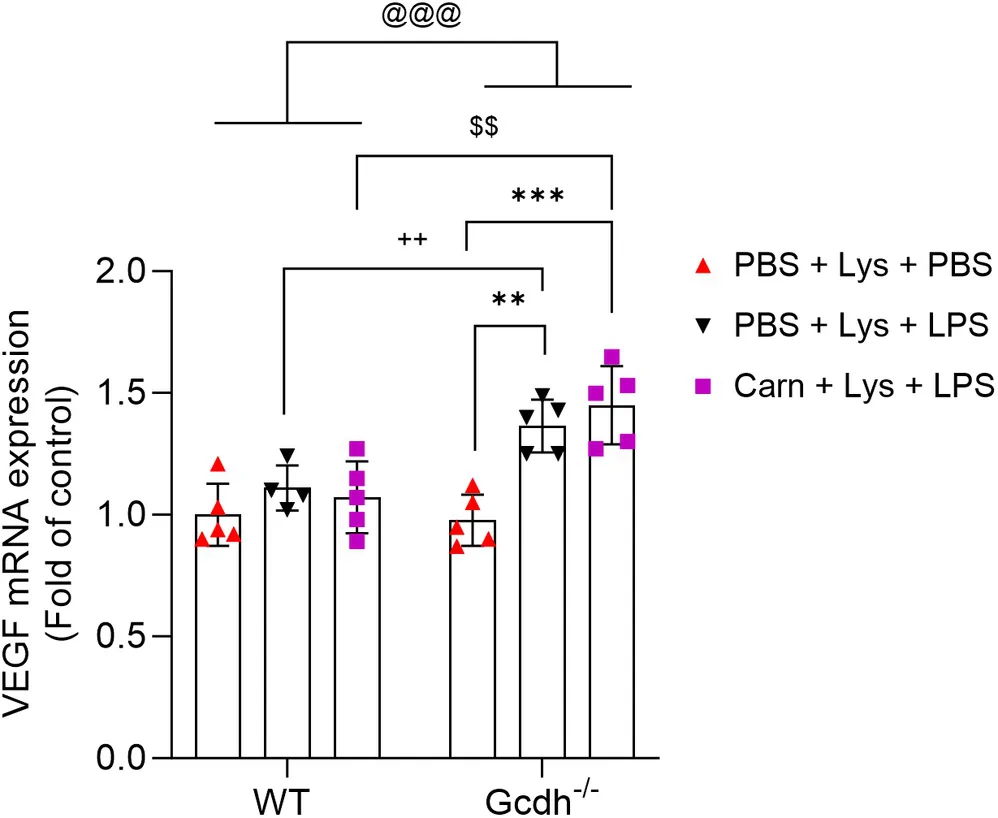
Vascular endothelial growth factor (VEGF) gene expression in the cerebral cortex of neonatal 7‐day‐old WT and *Gcdh* ^*−/−*^ mice. All animals were administered ip with Lys, whereas some with LPS and others with LPS plus Carn. Data are presented as mean ± SD, with *n* = 4–5 animals per group. Statistical analysis was performed using two‐way analysis of variance (ANOVA), followed by Tukey's post hoc test (**p < 0.01, ***p < 0.001 different from PBS + Lys + PBS; ^++^ *p* < 0.01 difference between WT and *Gcdh* ^*−/−*^ in LPS treated animals; ^$$^ *p* < 0.01 difference between WT and *Gcdh* ^*−/−*^ mice within the Carn + Lys + LPS treatment group; ^@@@^ *p* < 0.001 difference between WT and *Gcdh* ^*−/−*^).

We next evaluated whether the vascular endothelial growth factor (VEGF) gene expression was altered in LPS‐injected *Gcdh* ^*−/−*^ mice on PND7, by measuring its mRNA levels in the cerebral cortex of WT and *Gcdh* ^*−/−*^ mice. It can be seen in Figure 4 that VEGF gene expression was significantly increased in the cortex of LPS‐treated *Gcdh* ^*−/−*^ relative to WT mice, and that Carn did not prevent this increase (Treatment *F*_(2,23)_ = 13.78, *p* = 0.0001; Interaction *F*_(2,23)_ = 6.44, *p* = 0.0060; Genotype *F*_(1,23)_ = 18.35, *p* = 0.0003).

### 3.5 Carn Prevents LPS‐Induced Iba1 Staining Increase in Cerebral Cortex of 15‐Day‐Old WT Mice

**FIGURE 5:**
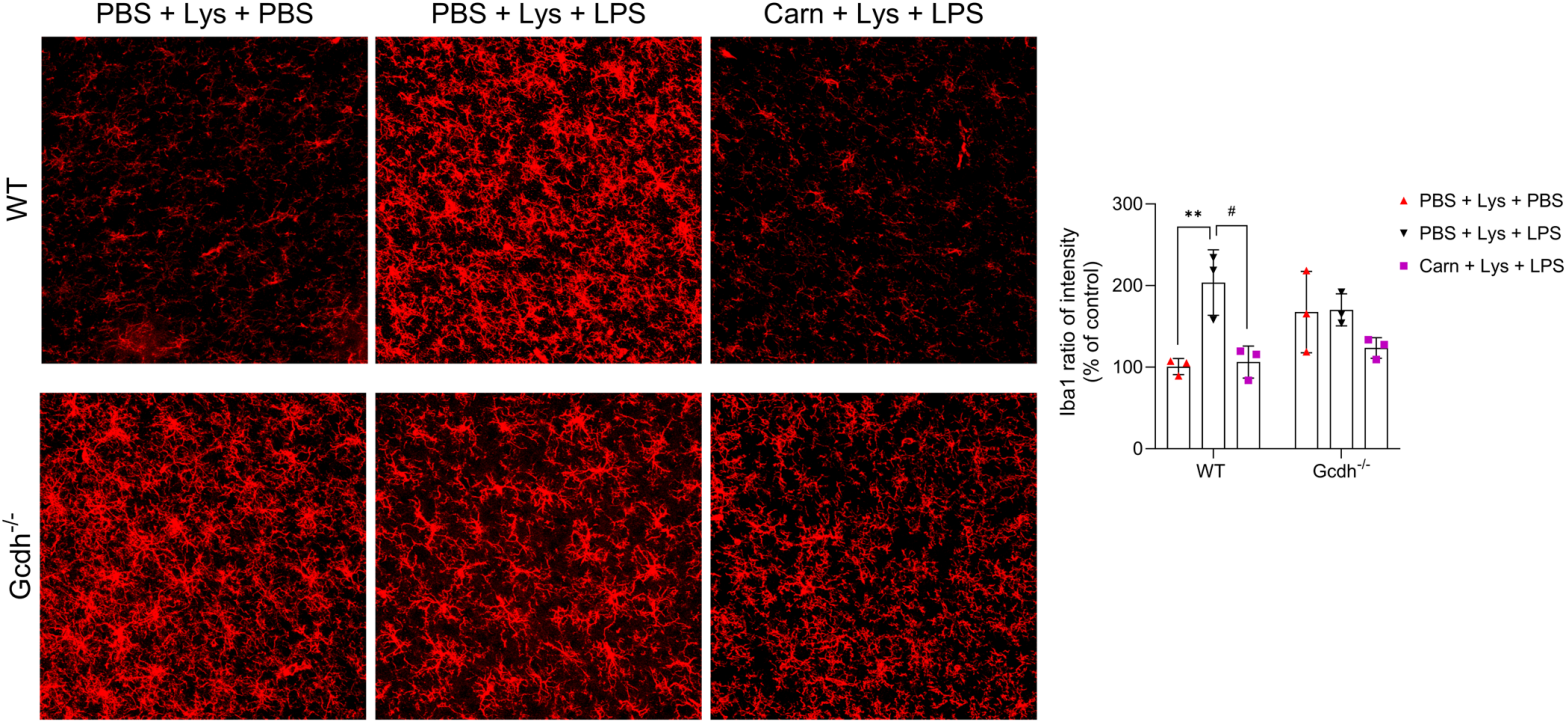
Confocal microscopy images showing immunohistochemical staining of the microglial protein Iba1 in the cerebral cortex of WT and *Gcdh* ^*−/−*^ mice on PND15. All animals were administered ip with Lys, whereas some with LPS and others with LPS plus Carn. Immunohistochemistry images were taken at 400× magnification and quantified using Fiji software based on the percentage of immunofluorescence intensity in the cerebral cortex. The values obtained were expressed as mean ± SD (two‐way ANOVA followed by post hoc Tukey's test), *n* = 3 animals per group (**p < 0.01 different from PBS + Lys + PBS; ^#^ *p* < 0.05 difference between PBS + Lys + LPS and Carn + Lys + LPS group).

Figure 5 shows a moderate nonsignificant increase (23%) of Iba1 in the cerebral cortex of *Gcdh* ^*−/−*^ relative to WT mice and that LPS treatment significantly increased this microglia activation biomarker in WT mice, which was totally normalized by Carn (Treatment *F*_(2,12)_ = 9.78, *p* = 0.0030; Interaction *F*_(2,12)_ = 4.42, *p* = 0.0364; Genotype *F*_(1,12)_ = 1.50, *p* = 0.2433).

### 3.6 Neuronal Viability Is Decreased in Cerebral Cortex of 15‐Day‐Old Gcdh^−/−^ Mice

**FIGURE 6:**
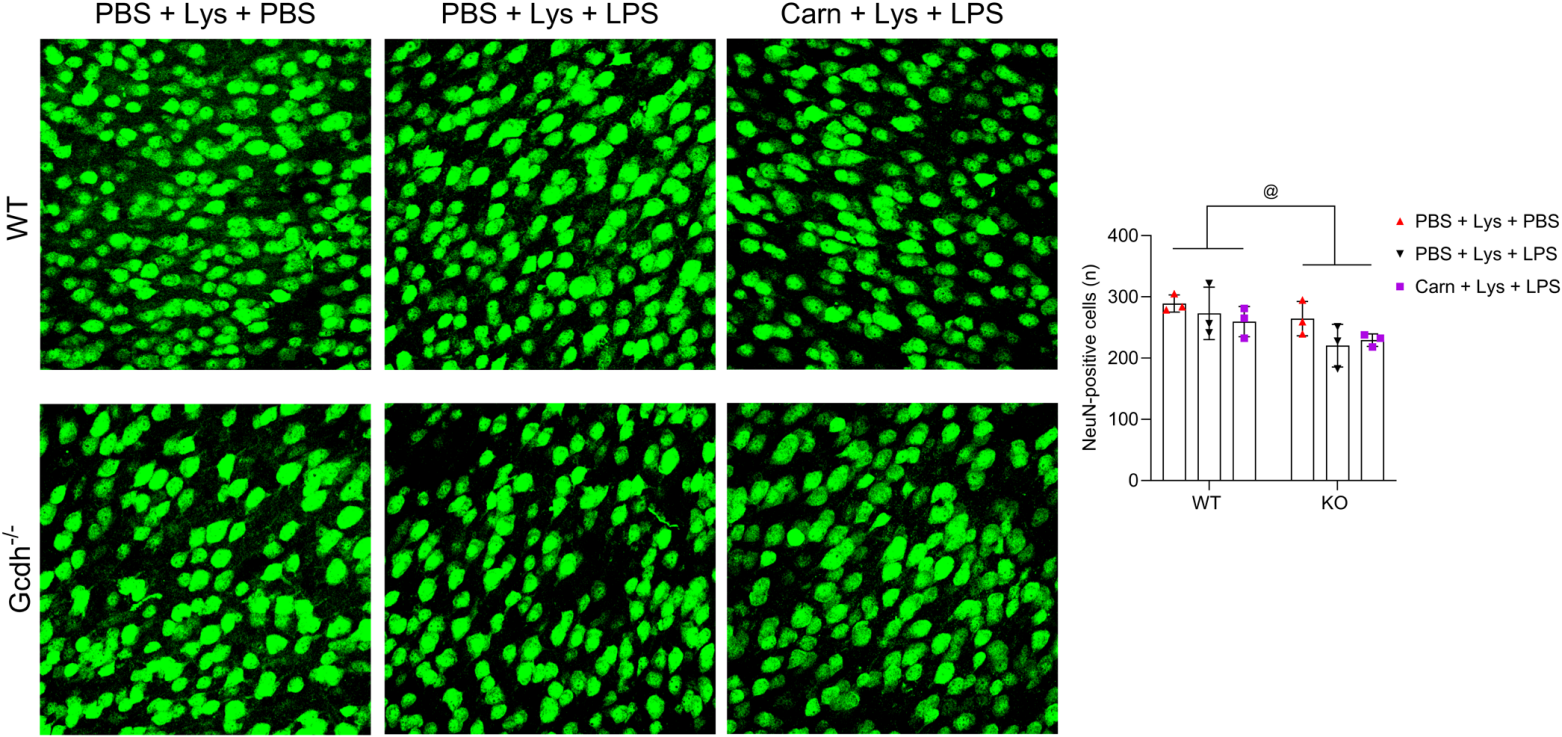
Confocal microscopy images showing NeuN immunohistochemical staining in the cerebral cortex of WT and *Gcdh* ^*−/−*^ mice on PND15. Immunohistochemical images of NeuN were visualized at 400× magnification, and the number of immunoreactive cells (viable neurons) was quantified using the Fiji software. The values are represented graphically as mean ± SD (two‐way ANOVA followed by post hoc Tukey's test), *n* = 3 animals per group (^@^ *p* < 0.05 difference between WT and *Gcdh* ^*−/−*^).

It can be seen in Figure 6 that the number of NeuN positive cells was mildly but significantly reduced in cerebral cortex of *Gcdh* ^*−/−*^ as compared to WT mice, and that LPS did not significantly alter neuronal viability. Carn also did not affect the number of NeuN positive cells in both WT and *Gcdh* ^*−/−*^ mice (Treatment *F*_(2,12)_ = 2.49, *p* = 0.1246; Interaction *F*_(2,12)_ = 0.42, *p* = 0.6667; Genotype *F*_(1,12)_ = 7.32, *p* = 0.0191).

### 3.7 Carn Prevents LPS‐Induced Increase of the Number of Vacuoles and Neurodegenerative Cells in Cerebral Cortex of 15‐Day‐Old Gcdh^−/−^ Mice

**FIGURE 7:**
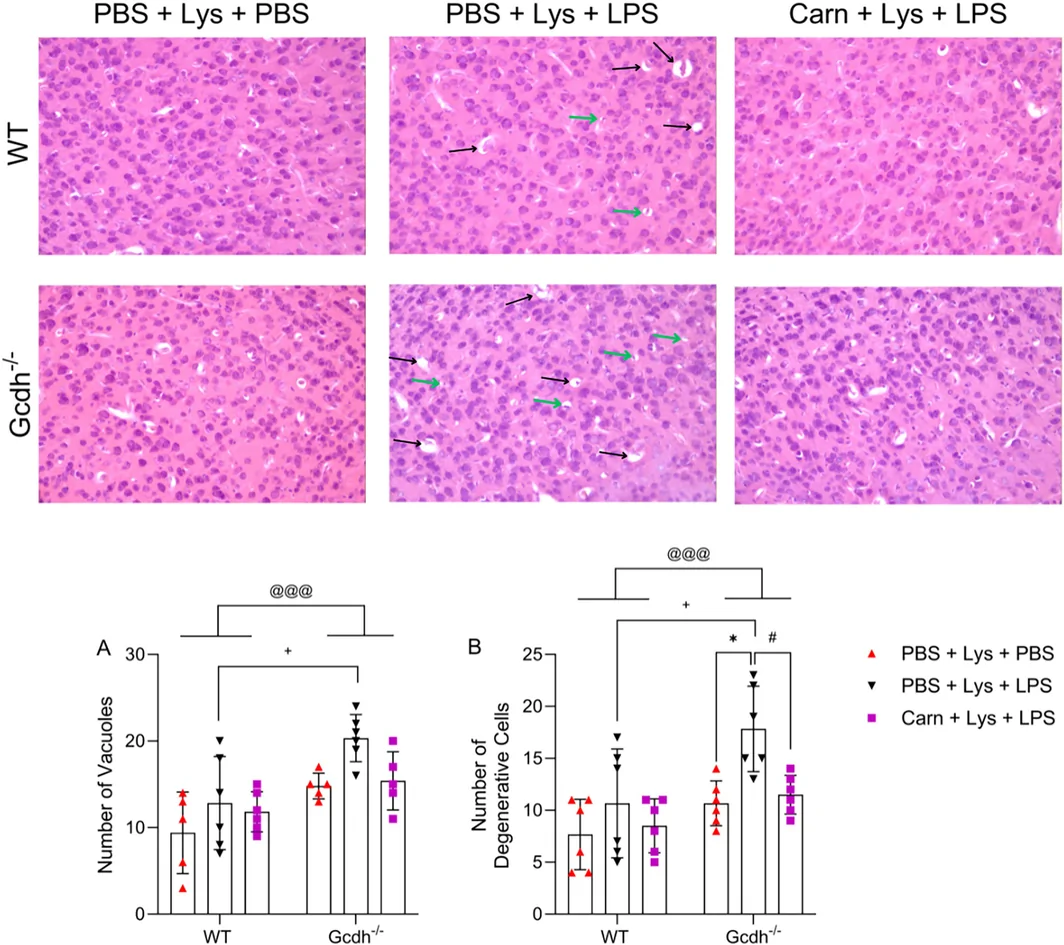
Vacuolation (A) (indicated by green arrows) and neurodegenerative cells (B) (indicated by the black arrows) on PND15 in the cerebral cortex of WT and *Gcdh* ^*−/−*^ mice. All animals were administered ip with Lys, whereas some with LPS and others with LPS plus Carn. Quantification of the number of vacuoles and degenerative cells was performed at 400× magnification. The results are presented as mean ± SD, with *n* = 5–6 animals per group. Statistical analysis was conducted using two‐way analysis of variance (ANOVA), followed by Tukey's post hoc test (*p < 0.05, different from PBS + Lys + PBS; ^#^ *p* < 0.05 difference between PBS + Lys + LPS and Carn + Lys + LPS; ^+^ *p* < 0.05, difference between WT and *Gcdh* ^*−/−*^ in LPS treated animals, ^@@@^ *p* < 0.001 difference between WT and *Gcdh* ^*−/−*^).

Figure 7 shows that the number of vacuoles (Treatment *F*_(2,27)_ = 4.45, *p* = 0.0215; Interaction *F*_(2,27)_ = 0.85, *p* = 0.4383; Genotype *F*_(1,27)_ = 18.90, *p* = 0.0002) and neurodegenerative cells (Treatment *F*_(2,30)_ = 7562, *p* = 0,0022; Interaction *F*_(2,30)_ = 1.47, *p* = 0.2456; Genotype *F*_(1,30)_ = 14.70, *p* = 0.0006) were higher in the cerebral cortex of *Gcdh* ^*−/−*^ relatively to WT mice, particularly in LPS treated animals, implying that neuroinflammation exacerbates neurodegeneration. Furthermore, Carn prevented neurodegeneration in the *Gcdh* ^*−/−*^ mice.

### 3.8 Carn Prevents LPS‐Induced Decrease of Body Weight and Righting Reflex Impairment in 10‐Day‐Old Gcdh^−/−^ Mice

**FIGURE 8:**
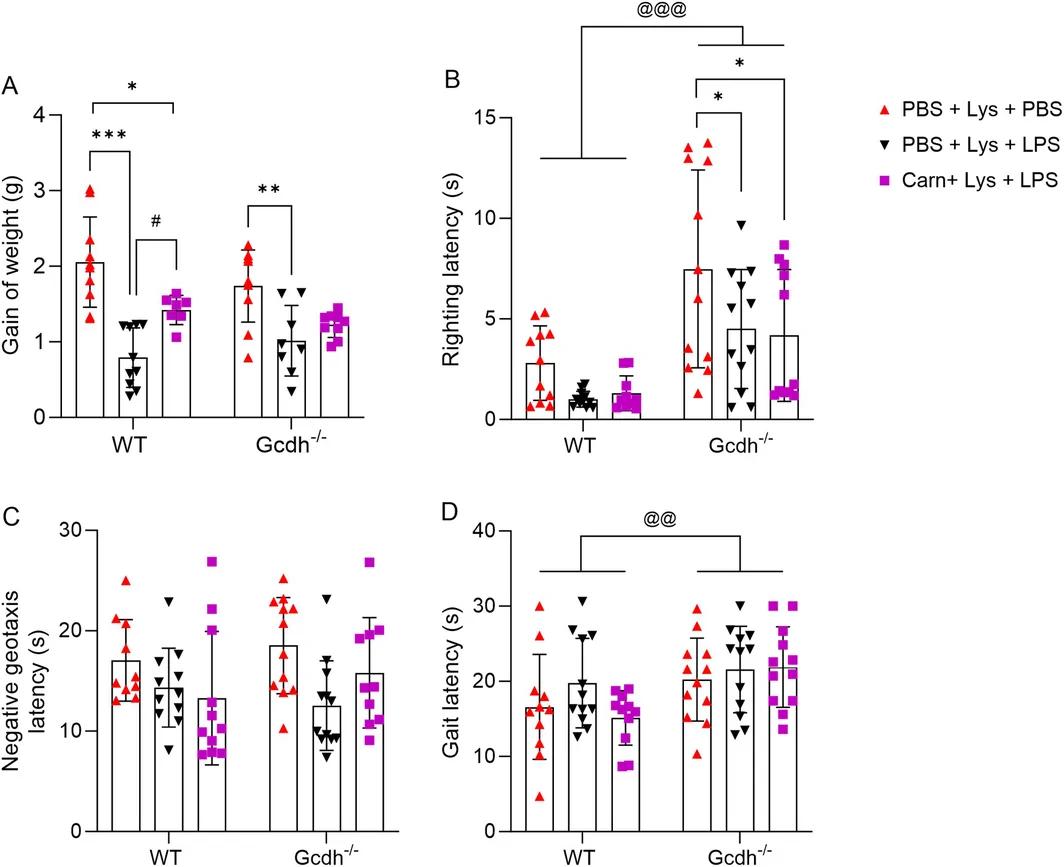
LPS‐induced neurodevelopmental delay in *Gcdh* ^*−/−*^ neonatal mice. LPS was injected on PND7 and markers of righting (B), negative geotaxis (C), and gait reflexes (D) were determined on PND10. Weight was also evaluated (A). All animals were administered ip with Lys, whereas some with LPS and others with LPS plus Carn. Two‐way ANOVA was performed followed by Tukey's post‐test, and the results are expressed as mean ± SD with *n* = 7–12 animals per group (*p < 0.05, **p < 0.01, ***p < 0.001 different from PBS + Lys + PBS; ^#^ *p* < 0.05 difference between PBS + Lys + LPS and Carn + Lys + LPS; ^@@^ *p* < 0.01 difference between WT and *Gcdh* ^*−/−*^, ^@@@^ *p* < 0.001 difference between WT and *Gcdh* ^*−/−*^).

Figure 8A shows that gain of weight was significantly reduced in LPS‐treated WT and *Gcdh* ^*−/−*^ mice and that Carn partly prevented this decrease (Treatment *F*_(2,48)_ = 26.24, *p* < 0.0001; Interaction *F*_(2,48)_ = 2.06, *p* = 0.1386; Genotype *F*_(1,48)_ = 0.705, *p* = 0.4052). It can also be seen in Figure 8B that the righting latency was higher in the *Gcdh* ^*−/−*^ mice, suggesting an impaired vestibular response (Treatment *F*_(2,61)_ = 5.14, *p* = 0.0086; Interaction *F*_(2,61)_ = 0.56, *p* = 0.5780; Genotype *F*_(1,61)_ = 26.94, *p* < 0.0001). Furthermore, *Gcdh* ^*−/−*^ mice exhibited mildly impaired gait performance (Figure 8D), indicating a subtle deficit in locomotor ability (Treatment *F*_(2,64)_ = 1.20, *p* = 0.3064; Interaction *F*_(2,64)_ = 1.16, *p* = 0.3193; Genotype *F*_(1,64)_ = 9.17, *p* = 0.0035). In contrast, negative geotaxis (Figure 8C) was similar in *Gcdh* ^*−/−*^ and WT mice (Treatment *F*_(2,60)_ = 4.55, *p* = 0.0145; Interaction *F*_(2,60)_ = 1.14, *p* = 0.3273; Genotype *F*_(1,60)_ = 0.36, *p* = 0.5529).

## 4 Discussion

The impact of the episodes of metabolic decompensation triggered by infectious diseases and biochemically characterized by high elevation of the toxic organic acids accumulated on GA I neuropathology is still very poorly known [11, 13, 17, 55, 56, 57, 58].

Although strict dietary Lys restriction and prompt and aggressive treatment during the encephalopathic crises prevent striatum degeneration in most patients, approximately one third of the “well‐treated” affected individuals exhibit acute or insidious striatal and cortical damage [18, 21] that could be a consequence of the acute episodes of metabolic decompensation and their deleterious effects. Therefore, further research into the mechanisms underlying brain injury in GA I, particularly those triggered by infectious diseases and the associated inflammatory responses, is essential for guiding the development of new, targeted therapeutic strategies for this disorder.

The establishment of the knockout murine model of GA I (*Gcdh* ^*−/−*^) [23] that was later improved by Lys or protein overload [24, 41] allowed a better model to investigate the pathomechanisms of brain damage in this disease. In this particular, mitochondrial dysfunction, oxidative stress, and disturbance of glutamatergic and GABAergic neurotransmission have been reported in the cerebral cortex and striatum of this genetic murine GA I model [25, 26, 30, 32, 33, 35, 37, 59, 60, 61, 62, 63].

In a previous study, Koeller and colleagues [23] verified that acute metabolic stress achieved by interferon or LPS administration that induced increased cytokine production in *Gcdh* ^*−/−*^ mice only caused a transient lower growth and no apparent neurologic dysfunction. However, these investigators did not overload the animals with lysine, which leads to GA and 3OHGA accumulation in the *Gcdh* ^*−/−*^ mice. Other investigators have shown that systemic administration of LPS stimulates a robust production of pro‐inflammatory cytokines in the brain by activation of signaling pathways such as NFκB and MAPK, as well as microglial activation [64, 65, 66, 67, 68]. Therefore, considering that practically nothing has been reported yet on the role of acute inflammation in the brain of neonatal *Gcdh* ^*−/−*^ mice submitted to acute Lys overload, in the present study WT and *Gcdh* ^*−/−*^ mice received ip injections of Lys and LPS to elicit higher levels of GA and 3OHGA and a systemic inflammatory response, respectively. Thereafter, we measured the mRNA expression of cytokines and other inflammatory biomarkers, as well as the signaling pathways involved in the inflammatory response, microglial activation, and neurodegenerative endpoints in the cerebral cortex of these animals. We also determined important landmarks of neuromotor development and whether Carn treatment could be neuroprotective against the neurochemical and neuromotor abnormalities observed.

We first observed a significant increase in TNF‐α, IL‐6, NFκB, and COX‐2 expression in *Gcdh* ^*−/−*^ mice relative to WT animals, indicating a basal pro‐inflammatory state. Furthermore, LPS elicited a strong activation of inflammatory signaling pathways in both genotypes, as reflected by the significant upregulation of NFκB, IκBα, and COX‐2. These findings indicate that LPS triggered innate immune signaling cascades in the brain through activation of the NFκB/IκB pathway, a key regulator of neuroinflammatory responses [69, 70]. Under physiological conditions, NFκB is retained in the cytoplasm through its association with inhibitory IκB proteins, particularly IκBα [71]. Upon LPS stimulation, IκBα undergoes phosphorylation and proteasomal degradation, allowing NFκB nuclear translocation and transcriptional activation of pro‐inflammatory genes, including cytokines, chemokines, and enzymes such as COX‐2 and nitric oxide synthase [67, 72].

Notably, the LPS‐induced increase in NFκB and COX‐2 expression was markedly greater in *Gcdh* ^*−/−*^ mice than in WT animals, indicating an exacerbated activation of the NFκB axis in the GA I model. In contrast, IκBα mRNA expression was similarly elevated in both genotypes following LPS administration, likely reflecting a compensatory negative‐feedback mechanism aimed at limiting excessive NFκB activity. Consistent with this interpretation, the enhanced NFκB activation observed in *Gcdh* ^*−/−*^ mice was accompanied by a greater induction of COX‐2, a well‐established NFκB‐regulated enzyme involved in prostaglandin synthesis and the amplification of neuroinflammatory responses in the CNS [72].

On the other hand, Lys administration alone increased TNF‐α mRNA expression in *Gcdh* ^*−/−*^ mice compared with WT animals, suggesting that the accumulation of lysine‐derived metabolites is sufficient to induce a low‐grade neuroinflammatory response. Although caution is warranted when extrapolating these findings to humans, they raise the possibility that neuroinflammation may already be present in patients with GA I under stable conditions and become exacerbated during infections or other inflammatory challenges.

In contrast to the pronounced effects of LPS on pro‐inflammatory mediators, HO‐1 immunocontent was not significantly altered by LPS in either genotype, although *Gcdh* ^*−/−*^ mice exhibited higher basal HO‐1 levels than WT animals. HO‐1 is a stress‐responsive enzyme regulated by redox‐sensitive transcription factors, particularly Nrf2 [73], and constitutes an important antioxidant and cytoprotective system in the CNS [74]. The elevated HO‐1 content in *Gcdh* ^*−/−*^ mice in the absence of LPS suggests a constitutive upregulation of stress defense mechanisms not dependent on acute inflammatory signaling. Given the enhanced NFκB activation and downstream COX‐2 expression observed in *Gcdh* ^*−/−*^ mice following LPS, the higher baseline HO‐1 might represent a compensatory antioxidant response aimed at counterbalancing chronic metabolic or redox stress in GCDH deficiency, potentially limiting the detrimental consequences of prolonged pro‐inflammatory signaling [75].

Carn, a natural compound found in red meat, fish, and dairy products that is synthesized at low amounts in the body, is crucial for transporting long‐chain fatty acids into mitochondria for ATP production [76]. Potent antioxidant and anti‐inflammatory properties have been recently demonstrated for Carn in various neurodegenerative diseases and inherited metabolic disorders [36, 38, 63, 77, 78]. Noteworthy, a recent work found increased levels of the proinflammatory cytokines IL‐1β, TNF‐α, and IL‐10 in the striatum of young adult *Gcdh* ^*−/−*^ mice fed a high Lys diet and, importantly, Carn was able to reduce the augmented levels of the pro‐inflammatory cytokine IL‐1β [44].

In the present work, Carn administration effectively mitigated LPS‐induced neuroinflammatory signaling in both WT and *Gcdh* ^*−/−*^ mice, restoring IL‐6, NFκB, and COX‐2 expression to baseline levels. This finding is consistent with meta‐analyses of randomized clinical trials showing that Carn reduces circulating IL‐6 and other markers of inflammation and oxidative stress [79]. Notably, Carn also normalized the exaggerated NFκB and COX‐2 responses observed in *Gcdh* ^*−/−*^ mice following LPS exposure. Similarly, acetyl‐L‐carnitine has been shown to suppress NFκB activation and attenuate oxidative damage in experimental models of LPS‐induced neuroinflammation [80]. Together, these findings indicate that Carn effectively counteracts neuroinflammatory signaling, even in conditions of increased susceptibility to inflammatory stimuli.

We also observed a moderate non‐significant increase in the gene expression of the microglial marker Iba1 in *Gcdh* ^*−/−*^ mice as compared to WT animals, and that LPS increased Iba1 expression in WT mice. Although this increase did not reach statistical significance, the data suggest a trend toward enhanced microglial activation in GCDH‐deficient mice. This pattern is consistent with our findings of TNF‐α expression and may be related to elevated levels of this pro‐inflammatory cytokine in *Gcdh* ^*−/−*^ mice even in the absence of LPS stimulation.

The neuronal marker NeuN was significantly reduced in *Gcdh* ^*−/−*^ mice, whereas vacuolation and the number of neurodegenerative cells were markedly increased, particularly following LPS administration, indicating enhanced neuronal damage associated with an exacerbated inflammatory response. These findings are consistent with the neurodegenerative phenotype reported in GA I patients [1] and *Gcdh* ^*−/−*^ mice [24, 41]. Importantly, Carn treatment prevented microglial activation, neuronal loss, vacuolation, and neurodegeneration, further supporting its neuroprotective effects. In parallel, VEGF expression was significantly increased in the cerebral cortex of LPS‐treated Gcdh^−/−^ mice compared with WT animals, suggesting activation of endogenous neuroprotective and repair mechanisms in response to acute injury. Given its role in promoting angiogenesis and improving oxygen and nutrient delivery to damaged tissues, VEGF may contribute to neuronal survival and tissue recovery following neuroinflammatory insults [81, 82, 83].

Given the pronounced neuroinflammatory profile and histopathological damage observed in *Gcdh* ^*−/−*^ mice, we investigated whether these alterations could impair early neuromotor development. Previous studies from our group showed that intracerebroventricular (icv) administration of GA induces sensorimotor deficits and delays visual cortex maturation in *Gcdh* ^*−/−*^ mice [34, 84]. Consistent with these findings, *Gcdh* ^*−/−*^ mice displayed impaired righting reflex and gait performance at PND10, indicating delayed sensorimotor development. Since the righting reflex is a reliable indicator of brain maturation and reflects the integrity of cortical and subcortical motor circuits [47, 52, 85], these deficits likely result from broader neurodevelopmental disturbances. Importantly, Carn treatment prevented cortical damage and normalized the righting reflex, suggesting that its neuroprotective effects contribute to functional recovery. Collectively, these findings support the notion that neuroinflammation and neurodevelopmental impairment are closely linked to the cortical neuropathology associated with GA I.

Although we did not measure free carnitine (C0) and glutarylcarnitine (C5DC) concentrations in blood of the WT and *Gcdh* ^*−/−*^ mice, we have previously described that C0 levels were decreased, whereas C5DC values were increased in the blood of *Gcdh* ^*−/−*^ mice relative to the *WT* mice, and that these differences were more evident after lysine supplementation [44]. In contrast, C0 and C5DC blood levels in *Gcdh* ^*−/−*^ and WT mice were reported to be similar in the original knockout model of GA I, although no data were presented [23]. These apparently conflicting results may be because the animals were not supplemented with lysine. In the present study, we used an acute systemic administration of Lys, which was previously shown to cause a high increase of GA concentrations (4 mM) in the cerebral cortex of *Gcdh* ^*−/−*^ mice [32]. It is therefore conceivable that the accumulation of GA in tissues and biological fluids in the genetic murine model supplemented by Lys may explain, at least in part, the differences in C0 and C5DC levels between the genotypes since Lys is easily converted to GA in tissues deficient in GCDH activity [86]. A point that should be emphasized is the need for a better and effective treatment for GA I patients, especially during early development, where brain vulnerability to the metabolic decompensation crises precipitated by infectious/inflammatory processes is critical for their survival and neurological complications [63, 87, 88, 89]. So, an appropriate management of the increased inflammatory response, which leads to cytokine release and immune cell mobilization, such as microglial cells, and consequent central nervous system damage seems to be critical in GA I treatment [90]. Increased cytokine production in the brain may cause deleterious consequences to brain normal functioning by disturbing neurotransmitter systems, impacting neurocircuits and altering blood–brain barrier permeability [91, 92]. A previous work with GA I patients out of crises revealed higher levels of cytokines in plasma, indicating a pro‐inflammatory state in these patients [63]. Our present results showing that LPS triggers the release of pro‐inflammatory cytokines in the brain support the presumption that neuroinflammation plays an important role in the pathogenesis of the neurological alterations following infectious/inflammatory processes in GA I patients.

The present findings provide experimental evidence that systemic LPS administration induces a robust cerebral inflammation in both WT and *Gcdh* ^*−/−*^ mice subjected to acute Lys overload, although the inflammatory response was much higher and associated with increased endpoints of neurodegeneration in the knockout mice. It is therefore concluded that neuroinflammation is deleterious to the GCDH deficient mice and may represent a relevant pathomechanism of brain injury in GA I, particularly during encephalopathic crises triggered by infections, which are commonly accompanied by increased systemic and neuroinflammatory processes. The observation that Carn prevented the LPS‐induced increase in most of the inflammatory biomarkers analyzed suggests that its anti‐inflammatory properties constitute an important therapeutic mechanism that may act synergistically with Lys dietary restriction in the clinical management of GA I patients.

## Author Contributions

Ediandra T. Castro and Moacir Wajner conceived and designed the study. Ediandra T. Castro generated and analyzed the majority of the experimental data. Ângela Zanatta and Ediandra T. Castro conducted animal handling, metabolite inoculation, and sample collection. Sâmela A. Cunha and Tailine Q. Tavares processed brain samples and prepared immunofluorescence slides, which were analyzed by Rafael T. Ribeiro and Ediandra T. Castro. Diorlon Nunes Machado performed histological sectioning, hematoxylin–eosin staining, vacuole quantification, and counting of degenerating cells. Manuela B. Marcuzzo and Ângela B. Zemniaçak carried out the Western blot analyses. Andrey V. S. Carvalho and Ediandra T. Castro performed the neurodevelopmental assessments and the corresponding data analyses. Rafael Teixeira Ribeiro, Ângela Zanatta, and Ediandra T. Castro conducted the RT‐qPCR experiments. Larissa D. Bobermin, Carlos A. Netto, Alexandre U. Amaral, Carmem R. Vargas, and Guilhian Leipnitz contributed to the formal analysis and critical review of the data related to RT‐qPCR, neurodevelopmental testing, immunofluorescence, neuropathology, and Western blot assays, respectively. Ediandra T. Castro wrote the first draft of the manuscript as part of her PhD thesis. Moacir Wajner performed the final formal analysis, acquired funding, administered the project, and critically reviewed and edited the manuscript. All authors contributed to data interpretation, critically revised the manuscript, and approved the final version.

## Funding

This work was supported by grants from Fundação de Amparo à Pesquisa do Estado do Rio Grande do Sul (FAPERGS) (grant number # 24/2551‐0001239‐7), Conselho Nacional de Desenvolvimento Científico e Tecnológico (CNPq) (grant number #402440/2021‐8), and Instituto Nacional de Ciência e Tecnologia Saúde Cerebral (INCT‐SC) (grant number #406020/2022‐1).

## Ethics Statement

All experimental protocols were approved by the Animal Experimentation Ethics Committee of Hospital de Clínicas de Porto Alegre (Protocol 20230483).

## Conflicts of Interest

The authors declare no conflicts of interest.

## Data Availability

The data that support the findings of this study are available from the corresponding author upon reasonable request.
